# International elite Para athletes’ perspectives on anti-doping: what works, what doesn’t and what’s next?

**DOI:** 10.1136/bmjsem-2025-002788

**Published:** 2025-12-12

**Authors:** Anna Qvarfordt, Göran Svedsäter, Kristina Fagher, Anna Bjerkefors, Sven Blomqvist

**Affiliations:** 1 Department of Occupational Health, Psychology and Sport Sciences, Faculty of Health and Occupational Studies, University of Gävle, Gävle, Sweden; 2 Department of Health Sciences, Lund University, Lund, Sweden; 3 Swedish School of Sport and Health Sciences, Stockholm, Sweden; 4 Department of Neuroscience, Karolinska Institute, Stockholm, Sweden

**Keywords:** Para-Athletes, Ethics, Doping, Questionnaire, Elite performance

## Abstract

**Objectives:**

Para athletes may face unique challenges in the anti-doping system, including issues of privacy, implementation, comprehension and accessibility. While previous studies have examined non-disabled athletes’ views, little is known about how international elite Para athletes perceive anti-doping procedures. The aim of this study was to increase the understanding of elite Para athletes’ experiences and perceptions of anti-doping procedures.

**Methods:**

Using a cross-sectional observational study design, data were collected via an online questionnaire distributed during major international Para competitions. A total of 109 elite Para athletes from 25 countries, representing three impairment groups—physical, visual and intellectual impairments—participated in the study. Data were analysed using descriptive statistics and non-parametric analytical tests.

**Results:**

Among elite Para athletes, 27% had never received anti-doping education during their sports careers, while those who had received such education felt more confident in staying updated on the prohibited list. Over half had never undergone doping testing, and over 70% had never submitted whereabouts information. Most athletes who had been tested found the procedure respectful and adapted to their impairment. However, 25% could not provide a urine sample without assistance from a doping control officer or another person. Furthermore, 40% of those who had submitted whereabouts information said updating it reduced their enjoyment of being an elite athlete.

**Conclusions:**

This study shows that many Para athletes, even at the elite level, have limited experience with key anti-doping procedures, highlighting a need for more inclusive, prioritised and accessible anti-doping efforts tailored to the needs of Para athletes.

**WHAT IS ALREADY KNOWN ON THIS TOPIC**

- Para athletes encounter unique challenges in anti-doping procedures, yet current frameworks seldom address their specific needs. Research exploring Para athletes’ perspectives on anti-doping systems remains scarce.

**WHAT THIS STUDY ADDS**

- This study shows that international elite Para athletes have limited experience with anti-doping education and procedures. It also highlights accessibility and procedural barriers that point to the need for more inclusive anti-doping strategies.

**HOW THIS STUDY MIGHT AFFECT RESEARCH, PRACTICE OR POLICY**

- These findings underscore the need for tailored anti-doping policies and further research to ensure equitable implementation across athlete groups, especially given that few Para athletes have been exposed to anti-doping procedures.

## Background 

 Para-sports have experienced significant growth in recent years, with an increasing number of athletes, sports and competitive levels.^1^ As more athletes enter the field, the pressure to perform and achieve excellence intensifies. Especially, the elite competitive environment on an international level can increase the temptation to use performance-enhancing drugs, which has been demonstrated among non-disabled athletes.^2 3^ To ensure fair play and the integrity of sports, the World Anti-Doping Agency (WADA) has established comprehensive anti-doping regulations that apply to all athletes, including those in Para sports.^4^ However, the enforcement of these policies in Para sports has been inconsistent, with studies indicating that Para athletes with physical, visual and intellectual impairment often face challenges that their non-disabled counterparts do not.^1^

Urine sampling procedures can be intrusive for all athletes, as they inevitably involve a loss of privacy. For athletes with physical or visual impairments, however, the situation may be particularly challenging since they may require assistance during the process, which can further compromise their privacy and independence.^5^ Similarly, athletes with intellectual impairments may find it difficult to understand and follow testing procedures.^6^ These unique challenges necessitate tailored approaches to anti-doping education and procedures to ensure that Para athletes are not unfairly disadvantaged. Recently, however, Boardley *et al*^7^ have drawn attention to the situation of Para athletes within the anti-doping system and developed recommendations for Para-specific anti-doping education, emphasising the need for tailored content, accessible formats and the inclusion of athlete-support personnel.

Still, there is very little information on how to adapt anti-doping procedures to Para athletes in WADA’s existing material related to anti-doping procedures and education, which potentially lead to mistrust and concerns about the fairness and effectiveness of the anti-doping system.

Over the past decade, an increasing focus on athletes’ perceptions of anti-doping procedures, particularly among non-disabled athletes, has contributed to a deeper understanding of their adherence to anti-doping regulations.^8^ However, research targeting Para athletes’ perceptions of anti-doping procedures is limited. A study by Qvarfordt *et al*^5^ found that a national cohort of Swedish elite Para athletes had a positive view on the anti-doping system. However, a significant portion had not received anti-doping education, and many had never undergone a doping control.^5^ Similar results were shown by Blank *et al*,^1^ who demonstrated that a notable portion of a European cohort of Para athletes experienced that an actual anti-doping control was their first contact with the system. Furthermore, the athletes perceived that doping is highly prevalent in Paralympic sports.^1^ Hurst *et al*^6^ used interviews to capture athletes with intellectual impairments’ perceptions of anti-doping, revealing difficulties in understanding testing procedures and a reliance on support personnel. Moreover, it has been shown that therapeutic use exemptions (TUEs) are more prevalent during the Paralympic Games compared with the Olympic Games,^9^ but still there is a lack of knowledge concerning Para athletes’ experience using TUEs, the prohibited list and whereabouts procedures.

Taken together, there is a lack of knowledge regarding how international elite Para athletes perceive the anti-doping system, and their own ability to comply with the rules given their impairment. Research exploring the experiences and perceptions of elite Para athletes across different impairment groups can inform more inclusive and effective anti-doping education and procedures. The overall aim of this cross-sectional study was to increase the understanding of elite Para athletes’ experiences and perceptions of anti-doping procedures. The specific aims were: (1) to explore Para athletes’ exposure to anti-doping education and procedures and (2) to describe the athletes’ perceptions of anti-doping procedures.

## Method

Given the limited existing empirical research in this area, the study was designed as an exploratory cross-sectional investigation aimed at generating initial insights into Para athletes’ perspectives on anti-doping. It followed the Strengthening the Reporting of Observational Studies in Epidemiology guidelines and was conducted with the support of the International Blind Sports Federation (IBSA), World Intellectual Impairment Sport (VIRTUS) and the International Wheelchair & Amputee Sports Federation (IWAS).

### Participants and data collection

The participants were recruited from three major international competitions in 2023–2024: the 2023 Global Games (VIRTUS) in Vichy, France, the 2023 IBSA World Games in Birmingham, England and the 2024 Malmö Open in Malmö (IWAS), Sweden. To be eligible for the study, the participants needed to have a physical, visual or intellectual impairment, have an international sports class according to the International Paralympic Committee (IPC)^10^ or VIRTUS classification system, compete at the international level and be able to respond to a survey in English, Spanish, Portuguese, Polish or French. These languages were selected as most relevant to the athlete population at the competitions studied, and translations were performed by native speakers familiar with parasport in consultation with the authors.

Data collection took place via an online questionnaire (SUNET Survey & Report) (online supplemental appendix A). Coaches were initially informed about the study through both written and verbal communication and subsequently asked their athletes whether they wished to participate. Those interested in participating received both verbal and written information, along with access to an online informed consent form. Once consent was given, they received a link to the full questionnaire. The questionnaire was based on a previous questionnaire used among non-disabled athletes^11^ and it was slightly revised to suit athletes with physical, visual and intellectual impairment. To ensure accessibility for athletes with various impairments, the questionnaire design was adapted to the Web Content Accessibility Guidelines^12^ and informed by previous own experiences of making similar surveys accessible to comparable populations.^13 14^ The questionnaire consisted of demographic questions, athletes’ experiences and perceptions of anti-doping policy, doping controls, whereabouts information, TUE and anti-doping education. Participants could skip items they did not wish to answer and were not required to respond to all questions. If needed, coaches were available to assist during survey completion, following standardised instructions to ensure neutral support. The survey has been tested by Para athletes with various impairments and was used in a national study in Sweden. Before the implementation of this study, Para athletes themselves participated in the development of the survey.^5^ The questionnaire follows the requirements for availability based on the European standard EN301549.

### Data analysis

Descriptive statistics were used to present demographics and athletes’ perceptions and experiences of anti-doping in Para sport. Subgroups were formed based on gender (male/female), years of elite-level competition (less than 5 years/more than 5 years), receipt of anti-doping education (yes/no) and type of impairment (physical, visual or intellectual). These subgroups were chosen as different types of impairment can pose unique challenges to anti-doping procedures, for instance requiring assistance during urine sampling, limiting the ability to follow testing procedures or affecting how regulations and education are perceived. To compare athletes’ scores across these subgroups, the Mann-Whitney U test or the Kruskal-Wallis H test was used (significance level of α=0.05). To account for multiple Mann-Whitney U tests, a Bonferroni correction was applied (α=0.0017; 0.05/30). All responses of ‘don’t know/can’t answer’ were excluded from the analysis. The effect size was calculated according to Rosenthal^15^ and over 0.5 is considered a large effect. Data were analysed using IBM SPSS Statistics, V.29 (IBM).

### Equity, diversity and inclusion statement

A project group was formed including Para athletes, representatives from the Swedish Paralympic Committee/Parasport Sweden, and researchers, each contributing their expertise to develop the project and survey.^16^ Focus was placed on athlete input in the initial phase, ensuring the questionnaire was adapted to the experiences of elite Para athletes and the context of Para sport.

## Results

### Athletes’ background and exposure to anti-doping procedures

A total of 109 Para athletes from 25 countries responded to the survey, representing the three impairment groups: 48.6% had an intellectual impairment, 27.5% a physical impairment and 23.9% a visual impairment (table 1).

**Table 1:** Characteristics of the study group (n=109)

Female class	44 (40.4%)
Male class	65 (59.6%)
20 years old or younger	18 (16.5%)
21–25 years old	27 (24.8%)
26–30 years old	29 (26.6%)
31–35 years old	11 (10.1%)
36–40 years old	10 (9.2%)
41–45 years old	7 (6.4%)
46–50 years old	6 (5.5%)
50 years old or older	1 (0.9%)
Australia	6 (5.5%)
Brazil	4 (3.7%)
Denmark	15 (13.8%)
Finland	6 (5.5%)
Hong Kong	3 (2.8%)
Iceland	3 (2.8%)
Italy	6 (5.5%)
Japan	18 (16.5%)
New Zealand	8 (7.3%)
Poland	7 (6.4%)
Portugal	11 (10.1%)
Spain	4 (3.7%)
Sweden	5 (4.6%)
Argentina, Canada, Egypt, Germany, Great Britain, Israel, Lithuania, Luxembourg, Norway, Romania, Turkey	one participant each
*Missing	2 (1.8%)
Athletic	16 (14.7%)
Basketball	8 (7.3%)
Cycling	3 (2.8%)
Goalball	17 (15.6%)
Indoor Soccer	3 (2.8%)
Judo	8 (7.3%)
Karate	4 (3.7%)
Swimming	11 (10.1%)
Table tennis	11 (10.1%)
Wheelchair basketball	6 (5.5%)
Wheelchair rugby	4 (3.7%)
Archery, Blind football, Boccia, Handball, Para bench press, Para badminton, Powerlifting, Riding, Rowing, Shooting, Sledge hockey	One participant each
*Missing	3 (2.8%)
Individual sport	63 (57.8%)
Team sport	43 (39.4%)
*Missing	3 (2.8%)
Years competing on top level	
Less than 5 years	38 (34.9%)
5–10 years	37 (33.9%)
More than 10 years	33 (30.3%)
*Missing	1 (0.9%)
Intellectual impairment	53 (48.6%)
Physical impairment	30 (27.5%)
Visual impairment	26 (23.9%)
Received anti-doping education	
No	29 (26.6%)
Yes	80 (73.4%)
Doping testing during sports career	
No	62 (56.9%)
Yes	46 (42.2%)
*Missing	1 (0.9%)
Doping-tested in competition last 12 months	
No	26 (23.9%)
Yes	20 (18.3%)
*Missing	63 (57.8%)
Doping-tested in out-of-competition last 12 months	
No	36 (33,0%)
Yes	10 (9.1%)
*Missing	63 (57.8%)
Granted a TUE for medical reasons	
Never	97 (89.0%)
One or two times	8 (7.7%)
Continuously	3 (2.8%)
*Missing	1 (0.9%)
Submitted whereabouts information	
No	78 (71.6%)
Yes	31 (28.4%)
Distribution of impairments among individuals needing daily assistance	(n=43)
Intellectual impairment	26 (60.5%)
Physical impairment	9 (20.9%)
Visual impairment	8 (18.6%)

* Missing indicates that the participant did not provide a response to that specific question in the questionnaire.

TUE, therapeutic use exemption.

59.6% competed in male categories, 64.2% had over 5 years of experience in high-level Para sport and 58.7% were aged 26 or older. Among the respondents, 26.6% had never received anti-doping education during their elite sports career. Over half (56.9%) had never undergone doping testing during their career, and in the past 12 months, 18.3% had been tested in-competition while only 9.1% had been tested out-of-competition. A majority of respondents had not submitted whereabouts information (71.6%) and had never been granted a TUE for medical reasons during their career (89.0%; table 1).

### Perceptions of doping controls and the prohibited list

Almost all (97.8%) of those who had undergone one or more doping tests felt positive that they had received adequate information about the procedure (71.7% strongly agreed; 26.1% agreed to some extent). In addition, 86.9% answered that the doping control was adapted to their impairment (39.1% strongly agreed; 47.8% agreed to some extent). The urine test procedure was also perceived as respectful by 97.8% (82.6% strongly agreed; 15.2% agreed to some extent). When asked if they could verify whether the doping officer followed the standard procedure, a relatively high number were unable to respond (19.6%) (table 2).

**Table 2:** Athletes’ experiences of doping control, the prohibited list, whereabouts reporting and technical solutions

	Strongly agree	Agree to some extent	Disagree to some extent	Strongly disagree	Don’t know/ can’t answer
Doping control and prohibited list
*The doping control personnel gives me the information I need during the doping control procedure (n=46)	33 (71.7%)	12 (26.1%)	–	–	1 (2.2%)
*I feel that the doping control situation is adapted to my impairment (n=46)	18 (39.1%)	22 (47.8%)	1 (2.2%)	2 (4.3%)	3 (6.5%)
*In urine doping test situations; the doping control personnel treats me with respect (n=46)	38 (82.6%)	7 (15.2%)	–	–	1 (2.2%)
*It is possible for me to control that the doping control officer acts according to international standard procedure (n=46)	22 (47.8%)	13 (28.2%)	2 (4.3%)	–	9 (19.6%)
It‘s hard for me to keep updated on the prohibited list (the doping list) (n=108)	27 (25.0%)	29 (26.9%)	20 (18.5%)	20 (18.5%)	12 (11.1%)
I think I have sufficient knowledge of the anti-doping rules to avoid unintentional doping (n=109)	55 (50.5%)	25 (22.9%)	10 (9.2%)	6 (5.5%)	13 (11.9%)
Whereabouts					
†It is easy to update whereabouts information (n=31)	10 (32.2%)	12 (38.7%)	5 (16.1%)	1 (3.2%)	3 (9.7%)
†The duty to provide whereabouts information reduces my joy of being an elite athlete (n=31)	9 (29.0%)	3 (9.7%)	6 (19.3%)	8 (25.8%)	5 (16.1%)
Technical solutions					
The technical solutions in various anti-doping procedures are adapted to my ability (n=106)	24 (22.6%)	30 (28.3%)	5 (4.7%)	2 (1.9%)	45 (42.4%)
*With today’s technical equipment, I can provide a urine sample myself without the help of the doping control officer/other person (n=46)	15 (32.6%)	23 (21.7%)	4 (8.7%)	8 (17.4%)	9 (19.6%)

* Includes only athletes who have undergone doping testing during their careers.

† Includes only athletes who have submitted whereabouts information during their careers.

When comparing subgroups, no significant differences were found in opinions about doping control based on gender, years on elite level, received anti-doping education and type of impairment (tables3  4). However, athletes who had received anti-doping education (Mdn=1) felt more confident in avoiding unintentional doping compared with those who had not received anti-doping education (Mdn=3), U=337.5, *z*=−4.280, p<0.001 (table 3).

**Table 3:** Comparative analysis by gender, experience and anti-doping education

	Groups (n)	Mean rank	Mann-Whitney U test	Z	P value	ES
Doping control and prohibited list						
*The doping control personnel gives me the information I need during the doping control procedure.	female (20) male (26)	23.71 23.31	255.00	−0.14	0.89	0.01
	less than 5 years (8) more than 5 years (38)	19.81 24.28	122.50	−1.09	0.28	0.16
	Received anti-doping education† yes (43) no (2)	–	–	–	–	–
*I feel that the doping control situation is adapted to my impairment	female (20) male (26)	21.93 24.71	228.50	−0.77	0.44	0.11
	less than 5 years (8) more than 5 years (38)	21.13 24.00	133.00	−0.6	0.55	0.09
	Received anti-doping education† yes (42) no (1)	–	–	–	–	–
*In urine doping test situations; the doping control personnel treats me with respect	female (20) male (26)	25.13 22.45	227.50	−1.09	0.27	0.16
	less than 5 years (8) more than 5 years (38)	22.31 23.75	142.50	−0.42	0.68	0.06
	Received anti-doping education† yes (43) no (2)	–	–	–	–	–
*It is possible for me to control that the doping control officer acts according to international standard procedure	female (20) male (26)	23.23 23.71	254.50	−0.13	0.9	0.02
	less than 5 years (8) more than 5 years (38)	19.69 24.30	121.50	−0.95	0.34	0.14
	Received anti-doping education† yes (36) no (1)	–	–	–	–	–
It’s hard for me to keep updated on the prohibited list (the doping list)	female (43) male (53)	51.35 46.19	1017.00	−0.94	0.35	0.1
	less than 5 years (34) more than 5 years (61)	45.41 49.44	949.00	−0.71	0.48	0.07
	Received anti-doping education yes (73) no (23)	52.48 35.87	549.00	−2.583	0.010	0.26
I think I have sufficient knowledge of the anti-doping rules to avoid unintentional doping	female (40) male (56)	42.15 53.04	866.00	−2.12	0.03	0.22
	less than 5 years (33) more than 5 years (62)	46.73 48.68	981.00	−0.37	0.71	0.04
	Received anti-doping education yes (76) no (20)	42.94 69.63	337.50	−4.280	<0.001‡	0.437
Whereabouts						
§It is easy to update whereabouts information	female (17) male (14)	13.50 19.04	76.50	−1.77	0.08	0.32
	less than 5 years (7) more than 5 years (24)	15.29 16.21	79.00	−0.25	0.8	0.05
	Received anti-doping education yes (24) no (4)	15.08 11.00	34.00	−0.99	0.33	0.19
§The duty to provide whereabouts information reduces my joy of being an elite athlete	female (17) male (14)	14.79 17.46	98.50	−0.84	0.4	0.15
	less than 5 years (7) more than 5 years (24)	11.79 17.23	54.50	−1.43	0.15	0.26
	Received anti-doping education yes (22) no (4)	14.77 6.50	16.00	−2.08	0.04	0.41
Technical solutions						
The technical solutions in various anti-doping procedures are adapted to my ability	female (43) male (63)	49.09 56.51	1165.00	−1.29	0.2	0.13
	less than 5 years (37) more than 5 years (68)	55.22 51.79	1176.00	−0.58	0.56	0.06
	Received anti-doping education yes (47) no (14)	31.54 29.18	303.50	−0.48	0.63	0.06
*With today’s technical equipment, I can provide a urine sample myself without the help of the doping control officer/other person	female (16) male (21)	19.94 18.29	153.00	−0.48	0.66	0.08
	less than 5 years (8) more than 5 years (29)	20.69 18.53	102.50	−0.52	0.6	0.09
	Received anti-doping education† yes (35) no (2)	–	–	–	–	–

* Includes only athletes who have undergone doping control during their career.

† No statistical analysis was conducted due to extreme skewness.

‡ Statistically significant difference (p<0.0017) according to Bonferroni correction.

§ Includes only athletes who have been required to submit whereabouts information.

ES, effect size.

**Table 4:** Comparison between classification groups

	Classification groups (n)	Mean rank	Kruskal-Wallis H	Df	P value	Effect size
Doping control and prohibited list						
*The doping control personnel gives me the information I need during the doping control procedure	intellectual (21) physical (12) visual (13)	22.67 26.38 22.19	1.23	2	0.54	nc
*I feel that the doping control situation is adapted to my impairment	intellectual (21) physical (12) visual (13)	24.74 24.13 20.92	0.82	2	0.66	nc
*In urine doping test situations; the doping control personnel treats me with respect	intellectual (21) physical (12) visual (13)	23.98 23.25 22.96	0.12	2	0.94	nc
*It is possible for me to control that the doping control officer acts according to international standard procedure	intellectual (21) physical (12) visual (13)	22.67 23.04 25.27	0.37	2	0.83	nc
It’s hard for me to keep updated on the prohibited list (the doping list)	intellectual (43) physical (28) visual (25)	38.19 61.61 51.56	13.3	2	<0.001†	see table 5
I think I have sufficient knowledge of the anti-doping rules to avoid unintentional doping	intellectual (53) physical (30) visual (26)	56.48 53.03 54.25	0.29	2	0.87	nc
Whereabouts						
‡It is easy to update whereabouts information	intellectual (18) physical (4) visual (9)	16.50 13.13 16.28	0.51	2	0.77	nc
‡The duty to provide whereabouts information reduces my joy of being an elite athlete	intellectual (18) physical (4) visual (9)	14.58 15.25 19.17	1.64	2	0.44	nc
Technical solutions						
The technical solutions in various anti-doping procedures are adapted to my ability	intellectual (50) physical (30) visual (26)	50.86 60.80 50.15	2.66	2	0.26	nc
*With today’s technical equipment, I can provide a urine sample myself without the help of the doping control officer/other person	intellectual (17) physical (9) visual (11)	18.15 22.83 17.18	1.71	2	0.43	nc

* Includes only those athletes who have taken a doping test during their career.

† Statistically significant difference (p<0.0017) according to Bonferroni correction.

‡ Includes only those athletes who have submitted whereabouts information during their career.

nc, not calculated (effect size only computed when significant group differences were found).

**Table 5:** Group differences in perceived difficulty updating on the prohibited list

	Classification groups (n)	Mean rank	Mann-Whitney U test	Z	P value	ES
It’s hard for me to keep updated on the prohibited list (the doping list)	II (43)–PI (28) II (43)–VI (25) PI (28)–VI (25)	29.09–46.61 31.09–40.36 29.50–24.20	305 391 280	–3.644 –1.950 –1.295	<0.001† 0.051 0.195	0.433 0.238 0.178

† Statistically significant difference (p<0.001) according to Bonferroni correction.

ES, effect size; II, intellectual impairment; PI, physical impairment; VI, visual impairment.

More than half of the respondents found it difficult to stay up to date with the prohibited list (table 2), and athletes with intellectual impairment (Mdn=2) found it significantly more difficult than those with physical impairment (Mdn=3), U=305, z=−3.644, p<0.001 (table 5). No other significant differences were found regarding opinions about the prohibited list across subgroups based on gender, years on elite level or received anti-doping education (table 3).

### Perceptions of the whereabouts system and TUEs

Only a minority of athletes (31 out of 109) had experience with the whereabouts system. Among them, 71% found it easy to update their information (32.2% strongly agreed; 38.7% agreed to some extent). A substantial proportion of athletes (38.7%) felt that updating whereabouts information reduced their enjoyment of being an elite athlete (29.0% strongly agreed; 9.7% agreed to some extent). A relatively larger proportion of respondents (16.1%) either could not or chose not to answer this question (table 2). No significant differences in opinions about the whereabouts system were found between subgroups based on gender, years at elite level, received anti-doping education and impairment groups (tables 3 and 4).

### Perceptions of technical barriers and support in doping control procedures

26.1% of Para athletes who had undergone doping testing were unable to provide a urine sample without assistance from a doping control officer or another person, despite the available technical equipment. When asked about technical solutions in various anti-doping procedures, 50.9% of Para athletes felt that these were adapted to their impairment, while 6.6% did not. A notable proportion of respondents were either unable or unwilling to respond to questions regarding technical solutions (table 2). No significant differences in opinions about technical solutions were found between subgroups based on gender, years at elite level, received anti-doping education or impairment groups (tables3  4).

## Discussion

The overall aim of this cross-sectional study was to increase the understanding of elite Para athletes’ experiences and perceptions of anti-doping procedures. The results showed that the Para athletes in this study had not encountered various anti-doping activities to the extent one might expect, given their participation at a high international level. Moreover, procedures within the anti-doping system posed various challenges for the athletes. Below, potential reasons and solutions for these findings are further discussed.

### Exposure to education and anti-doping procedures

The fact that more than half of the Para athletes had never undergone a doping test despite competing at a relatively high level should be considered problematic from an anti-doping point of view. Moreover, only 18.3% reported having been tested in-competition and 9.1% out-of-competition during the last 12 months, indicating that doping testing is rare throughout their careers and even more uncommon in recent times.

The proportion of athletes who had never been tested even exceeds the findings of a recent study on Swedish Para athletes, in which 48% had also never undergone a doping test.^5^ Furthermore, the results of this study indicate that only one third of the athletes are part of the whereabouts reporting system despite competing at elite level. According to the World Anti-Doping Code and the International Standard for Testing and Investigations (ISTI), international federations are required to establish a Registered Testing Pool (RTP) to determine which athletes qualify as ‘international-level athletes’.^17^ A concern is that there is a lack of clarity regarding how some Para sport organisations define ‘international-level athletes’, and how athletes are selected for inclusion in the RTP. According to ISTI, this information should be made public to ensure transparency for all stakeholders, including athletes and other anti-doping organisations (ADOs). On reviewing the websites of the respective federations responsible for the athletes in our study, it is evident that VIRTUS defines the highest-priority pool as established high-performance athletes, IWAS applies a similar definition, while IBSA has not published any information regarding its criteria for RTP inclusion. Subsequently, more clear definitions and transparency regarding RTPs are warranted within Para sports.

Another concern is that 27% of the participants in this study had not undergone anti-doping education, even though data were collected during major international competitions. WADA’s International Standard for Education clearly states that athletes should receive anti-doping education before participating in international competitions.^18^ For comparison to non-disabled athletes, a study of elite athletes subject to the USADA anti-doping rules reported that 100% of the 1398 athletes who participated had received education,^15^ although it should be noted that USADA operates within a higher WADA tier with extensive resources and strict education requirements.

The lack of education in our study may be related to the finding that one in five athletes in this study could not verify if the doping control was following standard procedures, which could be a concern for athletes’ rights and integrity. An important finding is that those who had undergone education felt more confident in avoiding mistake doping than those without education, highlighting the importance of education within the anti-doping system. WADA’s regulations state that an athlete’s first contact with anti-doping should occur through education. However, a previous study in Para sports has shown that many athletes (33%) reported that their first contact was during the actual doping control.^1^ Taken together, there is a need to intensify educational efforts about anti-doping within, and adapted to, Para sport. A very recent study reveals that the needs of Para athletes have not been sufficiently considered when designing anti-doping education,^7^ and there is a need for ADOs to adapt their education material to athletes with various impairments.

Regarding the prevalence of TUEs in this study (2.8% or 10.5% including occasional applicants), these results appear broadly consistent with previous research, although direct comparisons should be made with caution due to methodological differences. Vernec *et al*^9^ reported TUE prevalence rates between 0.7% and 1.1% among Olympic athletes, and from 2.2% to 4.1% among Paralympic athletes, based on TUEs valid specifically during the Games. Similarly, WADA’s Independent Observer estimated TUE prevalence among Paralympic athletes to range between 2.2% and 4.1% during competition periods.^19^,^22^ In contrast, our study assessed whether athletes had ever held a TUE, either continuously or on one or more occasions, regardless of timing in relation to major events. A recently published Swedish study found that approximately 17% of athletes had applied for and been granted a TUE at some point.^5^ The generally higher TUE prevalence among Paralympic compared with Olympic athletes likely reflects the greater need for prescribed medications related to the athletes’ specific impairment.^23^,^25^

### Perceptions of anti-doping procedures

Most athletes in this study reported positive attitudes towards the actual anti-doping procedure—findings consistent with previous studies on Swedish Para athletes.^5^ However, a concern is the athletes reporting not being able to provide a urine sample without the assistance of an additional person, which raises questions about integrity and privacy. Urine sampling in doping controls has previously been found to be problematic from an integrity perspective even in non-disabled athletes,^11 26^ and if multiple people are assisting during the sampling, the Para athlete may be exposed to situations lacking integrity and rule of law. For example, a blind athlete cannot control the name, amount of urine and whether it is the correct lid and jar. Adapting sampling procedures with technical aids could be a way to create equal conditions for all athletes. Qualitative studies including Para athletes could contribute to the development of adapted doping controls.

### Accessibility to the anti-doping system

One of the prerequisites for avoiding unintentional doping is that the athlete knows and can find out which substances are prohibited. More than half (51.9%) of all Para athletes in this study stated that it is difficult to stay updated on the prohibited list, and especially athletes with intellectual impairment found it difficult. A reason could be the low rate of education and also lack of accessible and understandable information. However, previous research has also shown that Olympic athletes report the same issue.^11^ Taken together, it could be recommended that information and reach to the Prohibited list are better adapted to athletes with various needs and abilities.

Furthermore, 19.3% of the participants in this study reported experiencing difficulties in keeping their whereabouts information updated. In addition, 38.7% of athletes stated that the requirement to report their whereabouts reduced their overall enjoyment of being elite athletes. These findings are consistent with previous research on Olympic athletes, where one-third have reported difficulties with the filing process,^11^ and one-third reported that the system detracted from their overall sporting experience.^11 27^ Subsequently, there is a need for exploring how the whereabouts system could be more user-friendly. Elite athletes are already exposed to a high amount of mental stress,^28^ and Para athletes may be exposed to additional stress related to their impairment and accessibility issues related to being an elite sports person and having an impairment.^25^

### Practical and policy implications

Para sport at international level is elite sport and should follow the same anti-doping measures as all sports.^29^,^31^ However, the results of the present study indicate that anti-doping efforts in elite Para sport are not being implemented to the extent required by the regulations. Furthermore, the results emphasise the necessity for tailored anti-doping strategies that consider the unique challenges faced by Para athletes. These include procedural adjustments for independence and privacy, increased access to education and the development of new accessible and technical solutions to enhance testing procedures. For example, a prohibited list for athletes with intellectual impairment could be valuable as well as the implementation of assistive urine collection tools (eg, tactile markings, privacy-preserving protocols). Furthermore, there is a need to ensure that the prohibited list and whereabouts platforms are accessible. By understanding and addressing these specific needs, anti-doping efforts can be more effectively implemented in Para sports, ensuring inclusion, fair play and athlete integrity.

Taken together, our findings suggest that Para sports may warrant increased attention within existing anti-doping frameworks. While risk assessment remains essential, we encourage stakeholders—including International Federations, National ADOs and WADA—to also account for inclusion, equity and the specific needs of athletes with impairments.

Even though the fundamental principle of anti-doping regulations is that they should apply to all athletes worldwide, the rules allow for adjustments to procedures. For example, the ISTI states that it is possible to modify the testing procedure for athletes with impairments, provided it does not risk ‘compromising the integrity of the Sample Collection Session’.^17^ According to the IPC, special consideration can also be given to so-called ‘protected persons’ in anti-doping contexts, a group that includes individuals with intellectual impairment.^32^ To what extent these modifications and considerations are applied in practice is unclear.

Despite the experienced challenges and limited testing and education experiences reported by many of the athletes, the responses nevertheless suggest a relatively high degree of conditional trust in the anti-doping system. While this should not be interpreted as representative of all Para athletes, the findings indicate that those who have interacted with the system generally felt informed and treated with respect, which may reflect an awareness among international anti-doping officers of Para athletes’ specific prerequisites.

### Limitations, strengths and future directions

This study has several limitations. First, the recruitment process relied on coaches to invite athletes to participate, which may have introduced selection bias and social desirability bias. The coach-based recruitment strategy may have favoured athletes who were more engaged or better supported institutionally. Although researchers were present on-site during competitions to answer questions from both athletes and coaches and provided standardised instructions to coaches to ensure neutral support, these risks cannot be fully excluded. Second, the cross-sectional study design entails a risk of recall bias. The relatively high proportion of missing responses to the questions concerning doping testing within the last 12 months may be due to the fact that few athletes had undergone doping testing during their careers and, therefore, chose not to respond. This creates some uncertainty in the findings related to these two items, and they should thus be interpreted with caution. In addition, the lack of qualitative data limited the possibility to explore participants’ perspectives in depth, as few responded to the open-ended questions. Third, the overall sample size was modest in absolute terms, and some major countries in Para sport did not participate. This underrepresentation may have influenced the findings, since anti-doping policies and educational processes could differ from those of the countries included. The sample was also skewed toward athletes from countries with stronger socioeconomic conditions, which limits the generalisability of the results. Finally, the inclusion of athletes with intellectual impairments raises validity concerns. However, as only athletes with mild intellectual impairment are eligible to compete at Virtus events, this subgroup was not fully representative.

Despite these limitations, this study with its exploratory nature also provides important strengths. While the overall sample was modest, it was relatively large compared with previous research in Para sport and included athletes from all impairment groups and a wide range of sports, both individual and team. Notably, athletes with intellectual impairments were included, a group often excluded from prior Para sport research. Furthermore, the questionnaire was developed and validated in collaboration with Para athletes with different types of impairments and was made available in several major languages, facilitating participation from non-English-speaking countries.

The decision to use a relatively long and conceptually rich questionnaire aimed to capture multiple facets of the studied phenomenon. While this design may have posed challenges for some respondents, potentially affecting data quality and interpretability, the study provides valuable initial insights into Para athletes’ perceptions and experiences. Future research could build on these findings by reflecting critically on survey design and content, exploring alternative methodological approaches such as qualitative or mixed-methods studies, and considering recruitment strategies to enhance inclusivity, particularly of athletes from low-resourced and middle-resourced countries. Such efforts would strengthen the evidence base and provide a more comprehensive understanding of anti-doping practices in Para sport.

### Conclusions

Overall, the results from this study indicate that many Para athletes have limited experience with key anti-doping procedures such as testing, whereabouts reporting and education. Given that a majority of the athletes in this study are elite athletes primarily from high-resourced settings, where anti-doping efforts are assumed to be well-supported, it is noteworthy that this population seems to be underprioritised in anti-doping measures. In summary, there is a collective need to improve anti-doping measures that are tailored to and accessible for Para athletes.

## Supplementary material

10.1136/bmjsem-2025-002788 online supplemental file 1

## Data Availability

Data are available on reasonable request.
