# Supplementary material for: International elite Para athletes’ perspectives on anti-doping: what works, what doesn’t and what’s next?
Source: BMJ Open Sport Exerc Med. 2025 Dec 12;11(4):e002788. doi: 10.1136/bmjsem-2025-002788 (PMC12706202; doi:10.1136/bmjsem-2025-002788)
Supplement: online supplemental file 1 [file bmjsem-11-4-s001.docx]

**Appendix A Questionnaire**

**I compete in** female class male class

I am

20 years old or younger 21-25 years old

26-30 years old

31-35 years old

36-40 years old

41-45 years old

46-50 years old

51 years old or older

My main sport is

The country/National team I compete for is

I have been competing in my sport at senior top-national/international level for

less than 5 years 5-10 years

more than 10 years

I compete in a Parasport for athletes with

intellectual impairment physical impairment visual impairment

If other, please specify

I need personal assistance in my daily living due to my impairment

yes no

Anti-doping education
Education is a component of the preventive work against doping in sports and anti-doping organizations at different levels are responsible to provide education.

I have received Anti-Doping education

never once

on more than one occasion

I think I have sufficient knowledge of the anti-doping rules to avoid unintentional doping

strongly agree

agree to some extent disagree to some extent strongly disagree

don't know/can't answer

Optional: I also would like to say this about anti-doping education (here you can write something that you find especially good or problematic with anti-doping education):

Doping controls
Doping control is one of the activities that Anti-Doping organizations (e.g. WADA, International Federation, National Anti-Doping Agency, Major Games Organizer) carry out in the work against doping. According to WADA regulations, athletes who compete at the international and national level may be tested anytime, anywhere.

I think that doping controls are an essential part of the work with keeping drugs out of my sport

strongly agree

agree to some extent disagree to some extent strongly disagree

don't know/can't answer

I believe that those who use prohibited substances can get away without being caught in doping tests

strongly agree

agree to some extent disagree to some extent strongly disagree

don't know/can't answer

I believe that the selection of athletes for doping controls is done in a fair way

strongly agree

agree to some extent disagree to some extent strongly disagree

don't know/can't answer

According to my knowledge, all athletes in my sport and at my level are met with the same extent/type of doping control in all countries

strongly agree

agree to some extent disagree to some extent strongly disagree

don't know/can't answer

It´s hard for me to keep updated on the Prohibited List (the doping list)

strongly agree

agree to some extent disagree to some extent strongly disagree

don't know/can't answer

I have undergone doping testing during my sports career

Yes No

During the last 12 months I have been tested in competition by an anti-doping organization

never once

2-4 times

5-9 times

10 times or more

During the last 12 months I have been tested out of competition by an anti-doping organization

never once

2-4 times

5-9 times

10 times or more

I feel like a suspected cheater when I am selected for a doping control

strongly agree

agree to some extent disagree to some extent strongly disagree

don't know/can't answer

It is OK when there is an unannounced doping control at my house

strongly agree

agree to some extent disagree to some extent strongly disagree

don't know/can't answer

The doping control personnel gives me the information I need during the doping control procedure

strongly agree

agree to some extent disagree to some extent strongly disagree

don't know/can't answer

I feel that the doping control situation is adapted to my impairment

strongly agree

agree to some extent disagree to some extent strongly disagree

don't know/can't answer

In urine doping test situations; the doping control personell treats me with respect

strongly agree

agree to some extent disagree to some extent strongly disagree

don't know/can't answer

It is possible for me to control that the doping control officer acts according to international

standard procedure

strongly agree

agree to some extent disagree to some extent strongly disagree

don't know/can't answer

The possibility for an Anti-Doping organization/test laboratory to store my test samples, violates my personal integrity

strongly agree

agree to some extent disagree to some extent strongly disagree

don't know/can't answer

Optional: I also would like to say this about doping controls (here you can write something that you find especially good or problematic with doping controls):

Whereabouts information
According to WADA regulations, an athlete in a registered testing pool is required to make a quarterly whereabouts filing that provides accurate and complete information about the athlete’s whereabouts during the forthcoming quarter, so that he/she can be located for testing at any time during that quarter.

I think that the whereabouts information system is an essential part of the work with keeping drugs out of my sport

strongly agree

agree to some extent disagree to some extent strongly disagree

don't know/can't answer

I believe that the whereabouts system is working properly in all countries

strongly agree

agree to some extent disagree to some extent strongly disagree

don't know/can't answer

I think that the whereabouts information system is a danger to an athlete’s privacy

strongly agree

agree to some extent disagree to some extent strongly disagree

don't know/can't answer

During my time as an athete I have been filing whereabouts information

yes no

It is easy to update whereabouts information

strongly agree

agree to some extent disagree to some extent strongly disagree

don't know/can't answer

The duty to provide whereabouts information reduces my joy of being an elite athlete

strongly agree

agree to some extent disagree to some extent strongly disagree

don't know/can't answer

People around me, such as family and friends, think that the whereabouts system restricts my social life

strongly agree

agree to some extent disagree to some extent strongly disagree

don't know/can't answer

Optional: I also would like to say this about whereabouts information (here you can write something that you find especially good or problematic with the whereabouts information

Therapeutic Use Exemption
According to WADA regulations, athletes with documented medical conditions requiring the use of a prohibited substance or a prohibited method may request a Therapeutic Use Exemption (TUE).

I have been granted a TUE for medical reasons

never

one or two times continuously

I think it is unfair that some athletes can be allowed to use otherwise banned substances

strongly agree

agree to some extent disagree to some extent strongly disagree

don't know/can't answer

I believe that all athletes applying for a TUE are treated in the same way

strongly agree

agree to some extent disagree to some extent strongly disagree

don't know/can't answer

Optional: I also would like to say this about therapeutic use exemptions (here you can write something that you find especially good or problematic with TUEs):

Anti-doping work in general

I think that the anti-doping work should develop in a way that there are

more anti-doping activities than today

the same level of anti-doping activities we have today less anti-doping activities than today

don't know/can't answer

I think that the current sanctions for anti-doping rule violations are

too mild

good as they are too harsh

don't know/can't answer

In the future, I think that we should deal with doping the following way:

it should remain prohibited

it should be liberalized under medical condition it should be allowed

don't know/can't answer

We must do whatever is necessary to prevent doping in sport even if it means restricting an athlete's private life

strongly agree

agree to some extent disagree to some extent strongly disagree

don't know/can't answer

The anti-doping activities have gone too far regarding the athletes private integrity

strongly agree

agree to some extent disagree to some extent strongly disagree

don't know/can't answer

I have thought about withdrawing from my sport at elite level since I think there is too much use of prohibited substances and methods involved

strongly agree

agree to some extent disagree to some extent strongly disagree

don't know/can't answer

I have thought about withdrawing from my sport at elite level since I think that the anti-doping

activities are too extensive

strongly agree

agree to some extent disagree to some extent strongly disagree

don't know/can't answer

The athletes should be more involved in the policy work (rules and regulations) around anti-doping

strongly agree

agree to some extent disagree to some extent strongly disagree

don't know/can't answer

Optional: I also would like to say this about anti-doping work (here you can write something that you find especially good or problematic with anti-doping efforts in general):

Technology and innovation

The technical solutions in various anti-doping procedures are adapted to my ability

strongly agree

agree to some extent disagree to some extent strongly disagree

don't know/can't answer

Here you can comment on the previous question

With today's technical equipment, I can provide a urine sample myself without the help of the doping control officer/other person

strongly agree

agree to some extent disagree to some extent strongly disagree

don't know/can't answer

Here you can comment on the previous question

Personally, I think there is a need for new technical solutions that can facilitate anti-doping procedures that I have come in contact with

yes no

You said that new technical solutions is needed. Can you describe what you are thinking of?

Optional: I also would like to say this about technical solutions in anti-doping (here you can write something that you find especially good or problematic with technical solutions):
